# Exceptional localization of intramuscular hemangioma: Insights from a 66-year-old case

**DOI:** 10.1016/j.radcr.2025.02.090

**Published:** 2025-03-15

**Authors:** Joud Boutaleb, Basma Beqqali, Sarah Loubaris, Manal El Beyeg, Ouijdane Zamani, Znati Kaoutar, Rachida Saouab, Jamal El Fenni

**Affiliations:** a Radiology Department, Mohammed Vth Military Hospital, Rabat, Morocco; b Anatomopathology Department, Chu Ibn Sina Hospital, Rabat, Morocco

**Keywords:** Case report, Hemangioma, MRI, Flexor hallucis longus muscle

## Abstract

Intramuscular hemangiomas are rare benign vascular tumors, accounting for less than 1% of all hemangiomas. They often present with subtle symptoms that gradually worsen over time. Magnetic resonance imaging (MRI) is the gold standard for diagnosis, providing detailed lesion characterization. Surgical resection remains the preferred treatment. We report the case of a 66-year-old patient with a painful swelling in the calf that had progressively increased in size over ten years, originating from the flexor hallucis longus muscle.

## Introduction

Intramuscular hemangiomas (IMHs) are benign vascular tumors that predominantly affect young adults, though they can also present in older individuals. IMHs represent approximately 0.8% of all hemangiomas and 7%-10% of benign soft tissue tumors. These lesions are classified into capillary, cavernous, and mixed subtypes, with the cavernous form being the most common in muscle tissue. Malignant transformation is exceedingly rare, with no reported cases of metastasis. The underlying pathophysiology involves aberrant endothelial cell proliferation and angiogenesis. Clinically, IMHs often present as painless, slow-growing masses; however, pain can develop depending on lesion size, location, and vascular engorgement. Magnetic resonance imaging (MRI) is the preferred modality for diagnosis. Management options include observation, sclerotherapy, embolization, and surgical excision, with complete resection being the most effective treatment. We report a rare case of an IMH within the flexor hallucis longus muscle in an elderly patient, emphasizing imaging findings and management strategies.

## Case presentation

A 66-year-old Moroccan woman presented with a progressively enlarging mass in the lower third of her left leg over the past 10 years. The lesion had recently become painful and exhibited a bluish discoloration. She denied any history of trauma. She also reported intermittent discomfort exacerbated by prolonged standing and walking. Her medical history included well-controlled hypertension, managed with lisinopril (10 mg daily). She had no history of diabetes, peripheral vascular disease, or other systemic conditions. She had no known vascular malformations or similar soft tissue lesions. Surgical history was notable for a cholecystectomy performed 15 years earlier for gallstones, without complications. Her family history was unremarkable, with no reports of similar tumors or vascular disorders.

On physical examination, the mass was firm, nonmobile, and measured approximately 10 cm in its longest dimension. It was tender to palpation, particularly during knee movement and resisted extension. A bluish discoloration was noted on the overlying skin, and the mass exhibited partial compressibility with limb elevation, suggesting a vascular etiology. No signs of distal ischemia, neurological deficits, or pulsatility were detected. There were no signs of systemic illness or constitutional symptoms.

Laboratory tests were within normal limits, including complete blood count (Hemoglobin: 12.5 g/dL [normal: 12-16 g/dL], White Blood Cell Count: 6,200 cells/μL [normal: 4,000-11,000 cells/μL]), liver and kidney function tests, and inflammatory markers (CRP: 0.5 mg/L, ESR: 12 mm/h).

**Fig. 1:**
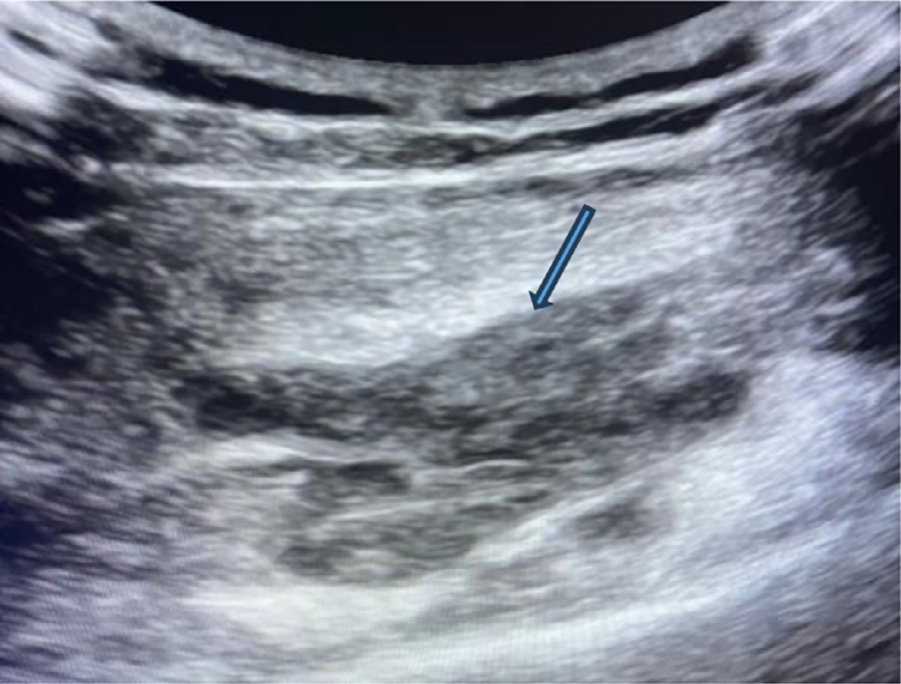
Ultrasound image depicting an oval, macro-lobulated, hypoechoic intramuscular mass with echogenic trabeculae.

**Fig. 2:**
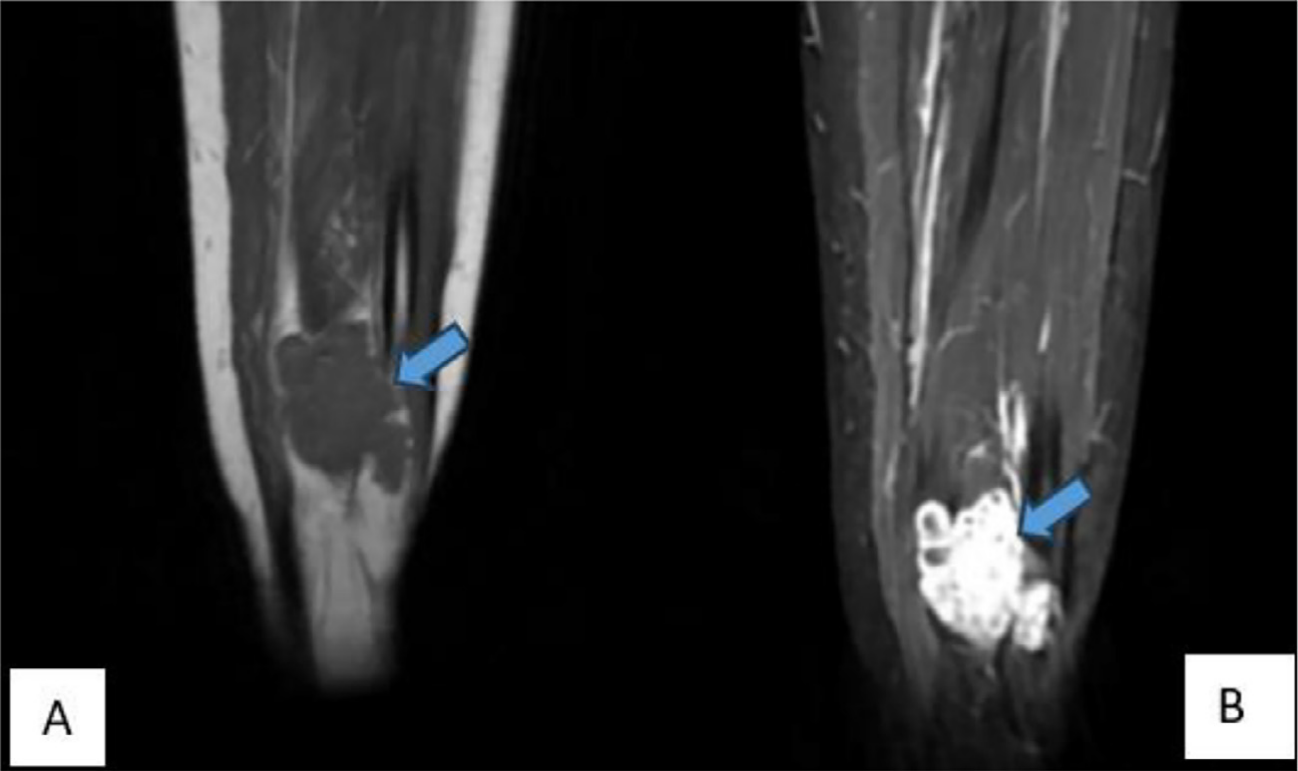
(A) Coronal T1-weighted MRI and (B) coronal STIR sequence demonstrating a well-defined, macro-lobulated intramuscular lesion within the flexor hallucis longus muscle. The lesion appears hypointense on T1-weighted images and markedly hyperintense on STIR sequences.

**Fig. 3:**
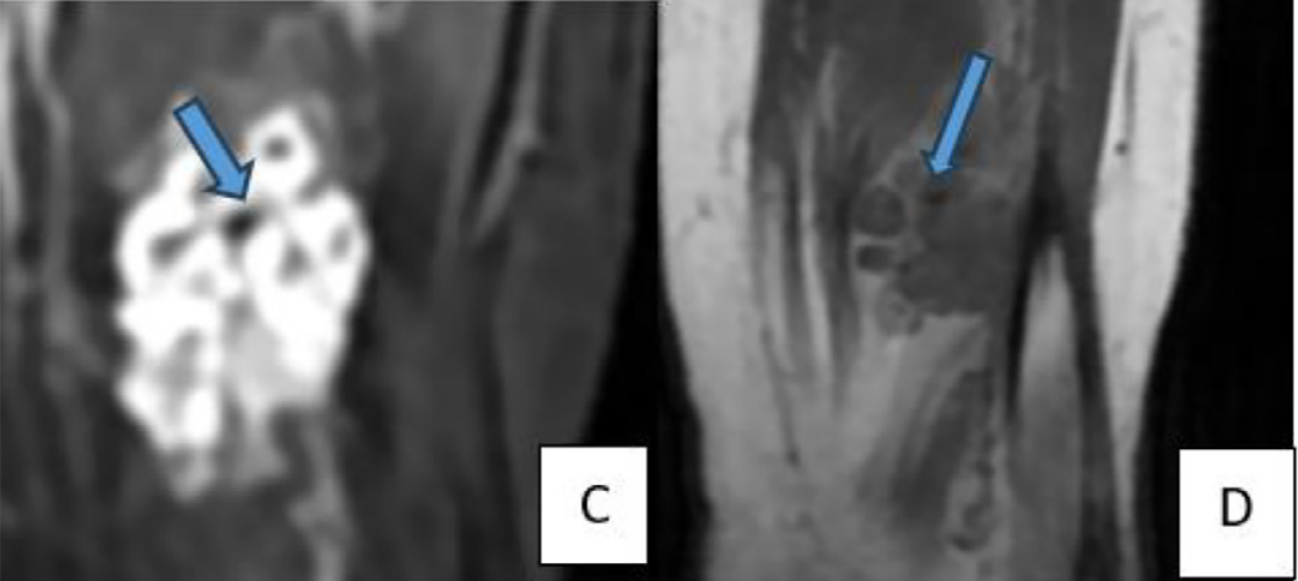
(C) Coronal STIR and (D) coronal T1-weighted sequences showing areas of internal calcification, appearing as signal voids, along with regions of T1- and T2-hypointensity.

**Fig. 4:**
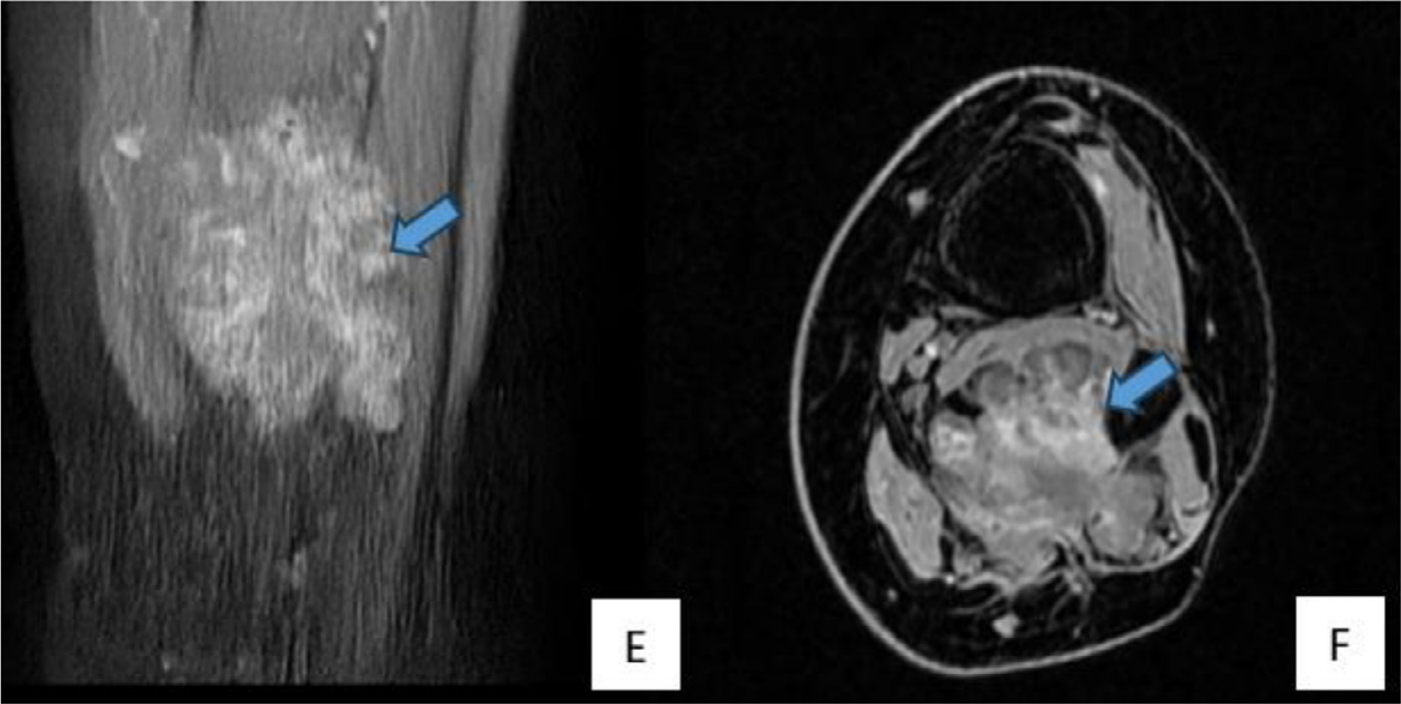
(E) Coronal and (F) axial postcontrast T1-weighted images demonstrating early and intense enhancement of the lesion following intravenous gadolinium administration, consistent with its vascular nature.

Ultrasound revealed an oval, macro-lobulated, hypoechoic intramuscular mass with echogenic trabeculae (Fig. 1). MRI of the lower leg showed a well-defined, macro-lobulated intramuscular lesion within the flexor hallucis longus muscle. The lesion displayed low signal intensity on T1-weighted imaging, heterogeneous hyperintensity on T2-weighted sequences, and high signal on STIR sequences (Fig. 2). Areas of internal calcification and regions of T1- and T2-hypointensity were noted (Fig. 3). Following gadolinium administration, the mass demonstrated early and intense enhancement (Fig. 4). The lesion was primarily vascularized by the peroneal artery, with no evidence of adjacent bone involvement.

**Fig. 5:**
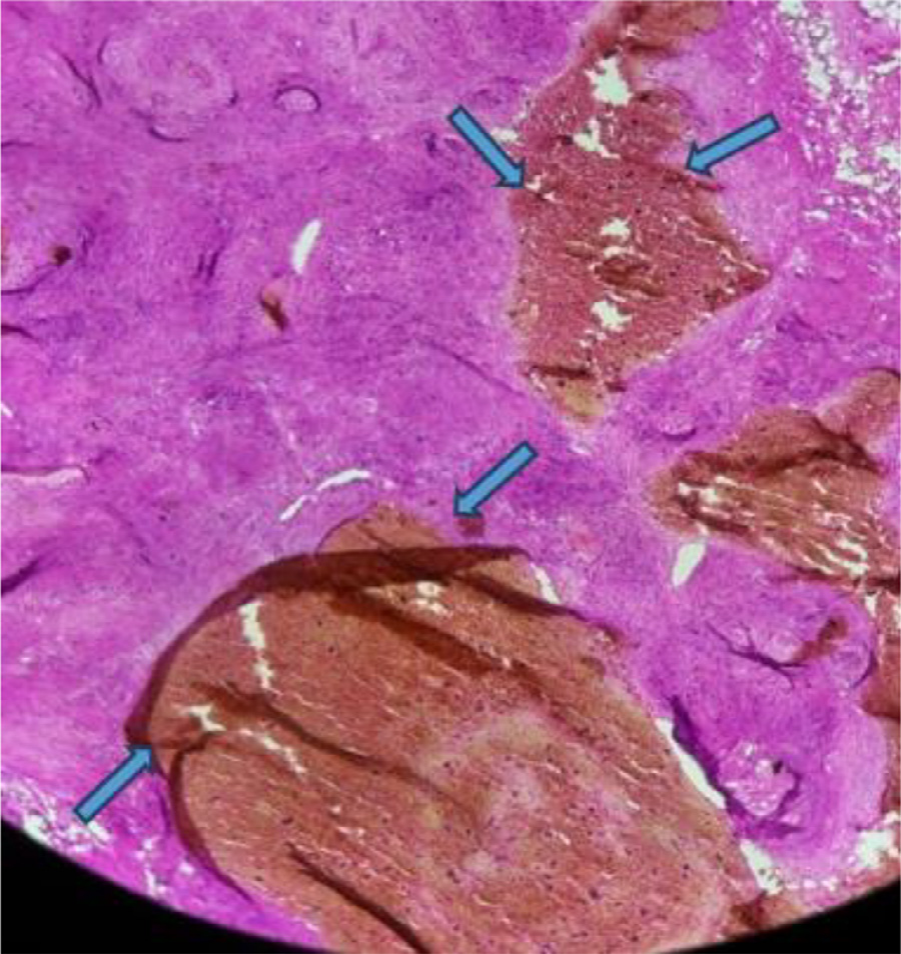
Histopathological examination confirming the diagnosis of hemangioma, revealing dilated, congested, and thin-walled blood vessels.

A percutaneous biopsy was performed for definitive diagnosis. Histopathological examination with hematoxylin and eosin (H&E) staining confirmed the presence of a benign vascular tumor. Immunohistochemical staining for CD31 and CD34 confirmed endothelial cell proliferation, further supporting the diagnosis of IMH. The lesion was composed of dilated vascular spaces lined by endothelial cells with eosinophilic cytoplasm and large, rounded nuclei. An inflammatory infiltrate with eosinophilic polymorphonuclear cells was also observed (Fig. 5). The patient was admitted to the trauma department for surgical resection. Preoperative planning involved collaboration with orthopedic and vascular surgery teams to optimize surgical access while preserving critical structures. The tumor was excised without complications. Postoperatively, the patient recovered well, with no immediate complications. She was discharged after a few days and reported significant pain relief at her follow-up visit. Wound healing was uneventful, and no recurrence has been observed during subsequent follow-ups

## Discussion

Intramuscular hemangiomas are rare, accounting for less than 1% of all hemangiomas (cutaneous, hepatic, cardiac, osseous, etc.) [5]. Their congenital origin is the most plausible hypothesis, as these lesions are typically present before the age of 30, with a higher incidence in females and a characteristic slow progression [6]. Allen and Enzinger reported a history of trauma in 5% of cases. Although any muscle can be affected, the quadriceps is the most frequently involved muscle in the lower extremity [1,6].

Hemangiomas are classified based on their histological characteristics and the size and structure of the blood vessels involved. The 3 main subtypes are [7]:

• Capillary hemangiomas: Composed primarily of small, thin-walled capillaries, these are the most common subtype and are often superficial, typically affecting the skin and mucous membranes. In intramuscular hemangiomas (IMHs), capillary components are less frequent but may be present, particularly in mixed-type lesions.
• Cavernous hemangiomas: These consist of large, dilated blood vessels forming cavernous spaces. They are typically deeper and present as a mass that can involve muscles or other deep tissues. Symptoms such as pain or swelling may occur due to the involvement of larger vascular spaces and local tissue compression. This subtype is more frequently seen in IMHs, particularly as part of mixed lesions.
• Mixed hemangiomas: These lesions contain both capillary and cavernous components, making them the most common subtype of intramuscular hemangiomas. Their mixed nature can complicate diagnosis and treatment, as the clinical behavior and vascular characteristics of the tumor vary depending on the proportions of each component.

Clinically, these lesions often present as painless, slowly enlarging masses; however, pain is the predominant symptom in approximately 60% of cases [1]. Key clinical features suggesting a vascular tumor include the presence of dilated superficial veins, tumor size reduction with limb elevation or pressure, and enlargement with physical activity due to increased blood flow. Large lesions may also present with a murmur or thrill [9].

The differential diagnosis includes arteriovenous malformations and other soft tissue masses, such as lipomas and soft tissue sarcomas (e.g., rhabdomyosarcoma, angiosarcoma). MRI is essential for distinguishing these entities, providing detailed information on the tumor's signal characteristics in T1- and T2-weighted sequences [[1], [2], [3], [4], [5], [6], [7], [8], [9], [10]]. X-rays may reveal rounded calcifications or phleboliths in up to 25% of cases, which are pathognomonic for slow-flow venous (cavernous) hemangiomas [11].

On MRI, intramuscular hemangiomas typically exhibit heterogeneous signal intensity on both T1- and T2-weighted sequences [12]. This heterogeneity results from the presence of various tissue components, including fat, smooth muscle, myxoid stroma, thrombus, and hemosiderin, each contributing different signal intensities. Thrombosis, fat, and slow-moving blood in cavernous spaces appear as areas of high signal intensity, whereas fibrous septa, rapid blood flow, calcifications, or hemosiderin appear as areas of low or absent signal [13]. Although MRI cannot reliably differentiate histological subtypes, it provides crucial information regarding tumor size, location, and vascularization. Intravenous gadolinium contrast typically results in significant enhancement, except in thrombosed vessels, where enhancement is reduced [14].

### Treatment

Treatment options depend on the hemangioma's location, size, and symptoms. Intramuscular hemangiomas pose a therapeutic challenge due to their deep tissue location and difficult surgical access. The treatment of choice is surgical excision, which is often curative [15]. A biopsy is typically performed to confirm the histological diagnosis [3]. Complete resection is recommended to minimize the risk of recurrence. Preoperative embolization may be indicated to reduce the risk of significant hemorrhage during surgery [15].

Other treatment options include sclerotherapy, corticosteroids, radiation therapy, and embolization:

• Sclerotherapy: Typically used for small-to-medium-sized lesions, sclerotherapy can reduce tumor size and alleviate symptoms such as pain and swelling. It is particularly beneficial when the hemangioma is near vital structures, making surgery risky. However, for larger or deeper intramuscular hemangiomas, sclerotherapy may be less effective and can cause adverse effects such as tissue necrosis or skin ulceration [16].
• Corticosteroids: Effective for cutaneous hemangiomas, corticosteroids may be used for intramuscular hemangiomas with inflammation or rapid growth. Oral or systemic steroid administration can induce regression, particularly in children. However, corticosteroids are often less effective in significantly reducing intramuscular hemangiomas [17].
• Radiation therapy: Considered for deep or inoperable hemangiomas, radiation may reduce tumor size and symptoms in some cases. However, its use remains controversial due to the risk of secondary malignancies, particularly in younger patients. Radiation is generally considered a last-resort option when other treatments fail [18].
• Embolization: Effective for large vascular lesions, especially those located in surgically inaccessible areas such as deep intramuscular hemangiomas. It is often combined with surgical resection for larger masses. However, embolization carries the risk of ischemia in surrounding tissues, and recurrence is possible if the entire vascular bed is not adequately occluded [19].

## Conclusion

Hemangiomas are common benign vascular tumors; however, intramuscular localization is rare, and involvement of the flexor hallucis longus muscle is exceptionally uncommon. MRI, in combination with plain radiography, remains the diagnostic gold standard for assessing the extent of the lesion and its relationship to adjacent structures in suspected cases of intramuscular hemangioma. This case highlights the importance of considering IMH in the differential diagnosis of deep soft tissue masses, particularly in atypical patient populations. A multidisciplinary approach is essential for optimizing diagnosis and treatment.

## Ethics approval

Our institution does not require ethical approval for reporting individual cases or case series.

## Patient consent

Written informed consent was obtained from a legally authorized representative(s) for anonymized patient information to be published in this article.
